# Supplementary material for: COVID-19 Vaccine Hesitancy among Population in Jazan Region of Saudi Arabia
Source: Healthcare (Basel). 2023 Nov 27;11(23):3051. doi: 10.3390/healthcare11233051 (PMC10706323; doi:10.3390/healthcare11233051)
Supplement: Supplementary file 1 [file healthcare-11-03051-s001.zip › healthcare-2620077-supplementary.pdf]

Table S1. Comparison between Middle School and High School education groups

Variables			Hesitant to receive COVID-19 vaccine	Willing to receive COVID-19 Vaccine	Total	*P*-value
Educational Level	Middle School	n (%)	11 (61.1%)	7 (38.9%)	18 (100%)	0.012
	High School	n (%)	43 (31.2%)	95 (68.8%)	138 (100%)	
	Total	n (%)	54 (34.6%)	102 (65.4%)	15600%)	

Table S2. Comparison between Middle School and University education groups

Variables			Hesitant to receive COVID-19 vaccine	Willing to receive COVID-19 Vaccine	Total	*P*-value
Educational Level	Middle School	n (%)	11 (61.1%)	7 (38.9%)	18 (100%)	0.047
	University	n (%)	156 (37.8%)	257 (62.2%)	413 (100%)	
	Total	n (%)	167 (38.7%)	264 (62.3%)	431 (100%)	

Table S3. Comparison between High school and University education groups

Variables			Hesitant to receive COVID-19 vaccine	Willing to receive COVID-19 Vaccine	Total	*P*-value
Educational Level	High School	n (%)	43 (31.2%)	95 (6.8%)	138 (100%)	0.161
	University	n (%)	156 (37.8%)	257 (62.2%)	413 (100%)	
	Total	n (%)	199 (36.1%)	352 (63.9%)	551 (100%)	
